# *Meloneis* Gen. Nov., a New Epipsammic Genus of Rhaphoneidaceae (Bacillariophyceae)

**DOI:** 10.1371/journal.pone.0032198

**Published:** 2012-03-19

**Authors:** Ioanna Louvrou, Daniel B. Danielidis, Athena Economou-Amilli

**Affiliations:** Department of Ecology and Systematics, University of Athens, Athens, Greece; J. Craig Venter Institute, United States of America

## Abstract

The diatom family Rhaphoneidaceae is characterized by high generic diversity and low species diversity with most genera known to have long stratigraphic ranges. The genera within this family are neritic marine, and mostly epipsammic. A new modern and epipsammic genus, *Meloneis* gen. nov., is described herein and is compared to all genera within Rhaphoneidaceae and especially to *Rhaphoneis* Ehrenberg s.l. Within *Meloneis* three new species and one variety are distinguished and described herein: *M. mimallis* sp. nov., *M. mimallis* var. *zephyria* var. nov., *M. akytos* sp. nov., and *M. gorgis* sp. nov.

## Introduction

The marine order Rhaphoneidales Round consists of two families, Psammodiscaceae Round & Mann in Round *et al.* 1990 [1] and Rhaphoneidaceae Forti 1912 [2]. The former includes only one living taxon: *Psammodiscus nitidus* (Gregory) Round & Mann. The latter includes the following thirteen genera: *Adoneis* G.W.Andrews & P.Rivera 1987 [3] with one living species and also one fossil species [4]; *Delphineis* G.W.Andrews 1977 [5] emend. G.W.Andrews 1981 [6] with many both living and fossil taxa; *Detonia* Frenguelli 1949 [7] with one living and one fossil species, recently transferred to *Neodetonia* S.Blanco 2011 [8] (see however [9]); *Dickensoniaforma* R.P.Scherer 1997 [10] with two fossil species; *Diplomenora* K.L.Blazé 1984 [11] with one species both fossil and living; *Drewsandria* P.A.Sims & R.Ross 1996 [12] with one fossil species; *Incisoria* Hajós, in Hajós & Stradner 1975 [13]) with three fossil species; *Lancineis* G.W.Andrews 1990 [14] with seven fossil species; *Neodelphineis* Takano 1982 [15] with two living species; *Perissonoë* G.W.Andrews & Stoelzel 1984 [16] with few, mainly living species; *Rhaphoneis* Ehrenberg 1844 [17] (see also [18], [19]) with many fossil and living species; *Sceptroneis* Ehrenberg 1844 [17] with more than 10 mainly fossil species requiring reinvestigation (see [1], [20]). Another genus (*Polygoneis*) of Rhaphoneidaceae was unofficially proposed as new in 2008 [21].

In this paper, a new genus of Rhaphoneidaceae, named *Meloneis*, is established, with four infraspecific taxa. Genus *Meloneis* is taxonomically distinct from the related genera *Rhaphoneis*, *Neodelphineis* and *Perissonoë*, or the superficially similar genera *Adoneis* and *Dickensoniaforma*. The new taxa of *Meloneis* were found as epipsammic around submarine hydrothermal vents of Milos Island (Greece). Another genus of Rhaphoneidaceae, i.e. *Detonia* Frenguelli, was taxonomically rearranged from epipsammic material of the same area ([22]). A catalogue of epilithic diatoms found distant of the vents is also available [23], together with euendolithic chlorophytes and cyanobacteria (see also [24]).

## Materials and Methods

Milos is an island in the middle of the Hellenic Volcanic Arc with some 35 km^2^ of geothermally active seabed [25], and Palaeochori Bay (24°31.00′E; 36°40.11′N) on the southeastern Milos is one of the most active geothermal submarine areas of the Aegean Sea [26]. The hydrothermal fluids of the submarine vents in Palaeochori are warm (max. 115°C), acidic (min. pH 3.54), highly saline, generally enriched over seawater in chloride, calcium, strontium, barium, sodium, potassium, lithium, silicon, iron, manganese, zinc, cobalt, lead, nickel, yttrium, vanadium but depleted in magnesium and sulphate [27]. The gases in these fluids contain mainly carbon dioxide up to 91.9%; methane, hydrogen, and hydrogen sulphide are also released at concentrations of up to 9.7%, 3.0%, and 8.1% respectively [25].

Sediment samples were collected from submarine hydrothermal vents in Palaeochori Bay during two multidisciplinary field trips of some European institutions in June 1996 and June 1997 in the framework of EU-funded programmes. The study area is open to the public and is not under any protection act, therefore, no specific permits were required for visiting the area, working in the field and collecting samples. Furthermore, the collection did not involve in any way endangered or protected species of any kind. Each vent is surrounded by three characteristic concentric zones of distinctive color precipitates [25]–[26], [28]–[29]: yellow (**Y**), white (**W**), and brown (**B**). The yellow color of the innermost zone results from sulfur condensing on sand grains; the nature of the white precipitates of the intermediate zone is a mixture of amorphous silica, Si nodules and hollow tubes containing elemental sulfur on the outer surfaces; the outermost brownish zone of the vent system consists of Mn-oxides which predominately precipitate at increasing distances from the vent outlet. Material was studied from all the three color precipitates in both collections (in 1996 at a depth of 7 m; and in 1997 at a depth of 4 m and 7 m), and additionally from a control site (**C**) outside of the vents (in 1997, at a depth of 4 m and 7 m). The collected samples were preserved in formaldehyde solution. Material was oxidized and slides were prepared for diatom analyses according to standard procedures [30]. Observations were made using Zeiss Axiolab microscope equipped with a Sony DSC-S85 digital camera and Jeol JSM-35 Scanning Electron Microscope equipped with Adda 3 Olympus Soft Imaging Solutions and Scandium Universal SEM Imaging Platform.

## Results

***Meloneis* Louvrou, Danielidis & Econ.-Amilli gen. nov.**

**Latin Diagnosis.** Genus novum Rhaphoneidacearum. Valvae planes facie, substrictis limbis, late lanceolatae usque lateribus laeviter convexis et apicibus leniter productis. Area axialis hyalina, distincta, angusta, recta adusque mediocriter lanceolata. Areolae circulares adusque ovatae velis rotoidibus dispositae in striis radialibus curvatis axialis areae. Striae transversae non frontales trans aream axialem. Una continua series areolarum super limbum observata; areolae limbi continuae transversis striis. Magna rimoportula unica locata inter areolas ultimae transversae striae ad ambo apices valvae. Ambo apices valvae pseudocellum duobus adusque nonnullis porellis minutis forma magis rotunda. Margo valvae facies decorata irregulari serie demissarum silicearum papillarum sinilium cupolae forma.

**Description.** New genus in Rhaphoneidaceae described herein. Valves with flat surface, shallow mantles, broadly lanceolate outline, smoothly convex sides and slightly produced apices. Axial area hyaline, distinct, narrow, linear to slightly lanceolate. Valve surface covered with round to ovate rota-type areolae, radiating in curved transverse striae from the axial area. Transverse striae not aligned across the axial area. A single continuous row of areolae runs along the mantle; mantle areolae aligned with the transverse striae. A single large rimoportula placed in-between the areolae of the last transversal stria at each valve end. A pseudocellus consisting of 2 to several fine pores in a rather circular pattern at each valve end. Edge of the valve face ornamented by an irregular row of low siliceous dome-like papillae.

GENUS TYPE: *Meloneis mimallis* Louvrou, Danielidis & Econ.-Amilli, sp. nov.

DERIVATION OF NAME: Me.lo.ne'.is. femin., N.G. fem. Melos = the name of the greek island *Mήλος* (also written Milos) and the greek suff. neis, from N.G. *ναũς* (gen. νηός) = boat.

***Meloneis mimallis* Louvrou, Danielidis & Econ.-Amilli spec. nov. var. *mimallis***

**Figure 1:**
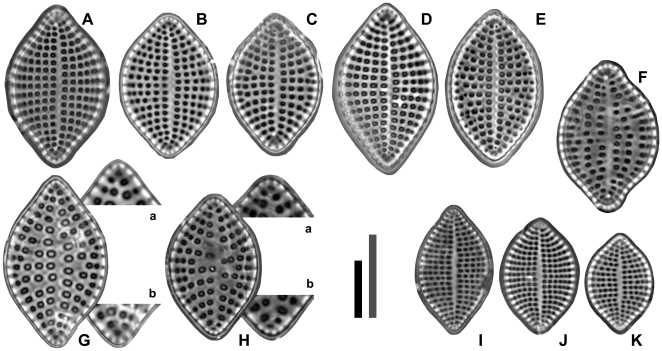
Species of *Meloneis* gen. nov. under LM. **Figure 1A** **.** *Meloneis mimallis* sp. nov., type of the genus. **Figures 1B–E** **.** Morphological variation of *M. mimallis* in the type locality. **Figure 1F** **.** *M. mimallis* var. *zephyria* var. nov.; the type. **Figure 1G** **.** The type of *M. akytos*, with two pseudocelli consisting of 3 & 4 pores respectively; note the robust areolae, and the distant striae. Magnified valve apices in Figs. 1Ga and 1Gb. **Figure 1H** **.** A valve of *M. akytos* with 2 pores per each pseudocellus, otherwise morphologically similar to the type. Magnified valve apices in Figs. 1Ha and 1Hb. **Figures 1I–K** **.** Morphological variation of *M. gorgis* sp. nov.; figure 1I represents the type. [Scale bars = 10 µm; the grey bar only for Figs. 1Ga, 1Gb, 1Ha, 1Hb).]

**Figure 2:**
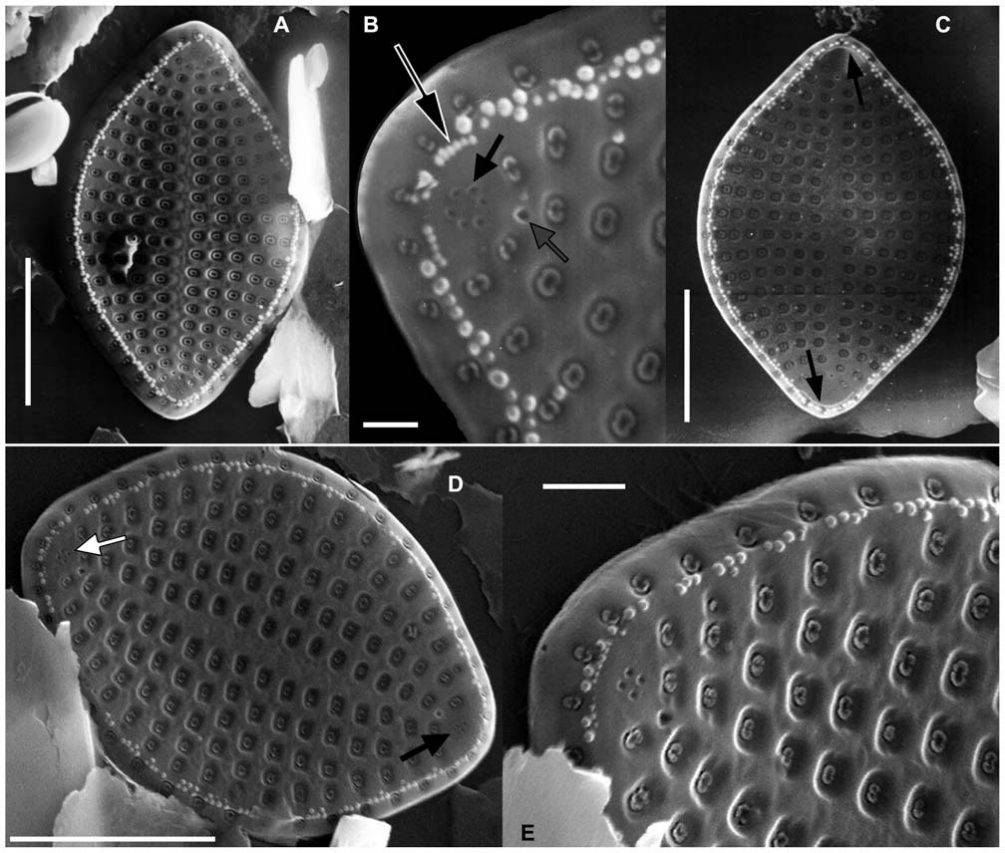
External valve views of *Meloneis mimallis* sp. nov. under SEM. **Figure 2A** **.** *M. mimallis* with two pseudocelli consisting of 5 pores (lower valve apex) & 6 pores (upper valve apex); note the two sizes of edge papillae, the larger ones usually in groups of 3–4 between the areolae rows. **Figure 2B** **.** Detail of the valve apex showing the circular pattern of pseudocellus consisting of 6 pores (black arrow); grey arrow indicates the external opening of the rimoportula; black arrow with white outline points to the extra incomplete row of small papillae (see also the complete rows in Fig. 2C). **Figure 2C** **.** Note the presence of an extra short continuous row of fine papillae at each pole (arrows) (cf. Fig. 2B). **Figure 2D** **.** A specimen with two pseudocelli, consisting of 3 pores (black arrow) & 4 pores (white arrow). **Figure 2E** **.** Detail of the valve end showing the round to ovate rota-type areolae with central pits. [Scale bars: Figures 2A, C, D = 10 µm, Figure 2B = 1 µm, Figure 2E = 2 µm].

**Figure 3:**
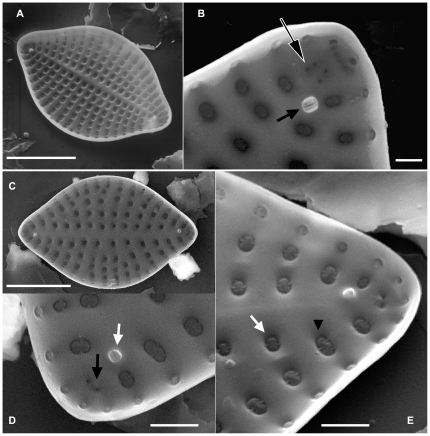
Internal valve views of *Meloneis mimallis* sp. nov. and *Meloneis akytos* sp. nov. under SEM. **Figure 3A** **.** *M. mimallis* with diagonally positioned rimoportulae in relation to the apical axis. **Figure 3B** **.** Detail of the apex showing the elongated slit-like internal opening of the rimoportula (black arrow), and the pseudocellus consisting of 4 fine pores (black arrow with white outline). **Figure 3C** **.** A specimen of *M. akytos* with distant striae and diagonally positioned rimoportulae in relation to the apical axis. **Figure 3D** **.** *M. akytos* showing the elongated slit-like internal opening of the rimoportula (white arrow), and the pseudocellus with 2 fine pores (black arrow). **Figure 3E** **.** Tilted specimen of *M. akytos*; note the two (white arrow) or three (black arrowhead) struts of the rotae in the areolae. [Scale bars: Figures 3A, C = 10 µm, Figure 3B = 1 µm, Figures 3D, E = 2 µm].

Figures 1A–E, 2A–E, 3A–B

**Latin Diagnosis.** Valvae planes facie, substrictis limbis, late lanceolatae, usque lateribus laeviter convexis et apicibus minime productis et rotundatis; 21.5–29 µm longitudine, 14.5–18.5 µm latitudine. Area axialis hyalina, distincta, angusta, recta adusque mediocriter lanceolata. Areolae magnae circulares adusque ovatae velis rotoidibus dispositae in striis radialibus curvatis axialis areae. Areolae in striis radialibus, in medio (7.5) 8–8.5 (9)/10 µm, in margine 6–7 (7.5)/10 µm, non frontales trans aream axialem. Areolae etiam dispositae in lineis minime curvatis in longitudinem, 6–7.5–(8)/10 µm in medio. Paucae breves striae marginales raro praesentes inter perfectas strias. Una continua series areolarum trancurrit limbum valvae; areolae limbi continuae transversis striis. Rimoportulae, una in singulam apicem, locatae in diagonio vel in utram partem axis apicalis. Rimoportulae rotundo externali porello, et parte interna longa unica caesa. Valvae pseudocello extenuato ambobus apicibus. Pseudocelli se constant tribus adusque septem porellis minutis per valvam. Margo valvae facies decorata irregulari serie demissarum silicearum papillarum sinilium cupolae forma; una series praeterea subtilarum papillarum solum in polis.

**Description.** Valves with flat surface, shallow mantles, broadly lanceolate outline, smoothly convex sides, and rounded slightly produced ends; length 21.5 to 29 µm, width 14.5 to 18.5 µm. Axial area hyaline, distinct, narrow, linear to slightly lanceolate. Valve surface covered with large circular to ovate rota-type areolae radiating in curved transverse striae from the axial area. Transverse striae radial, in the middle (7.5) 8–8.5 (9)/10 µm, at the margin 6–7 (7.5)/10 µm, not aligned across the axial area. Areolae arranged also in slightly curved longitudinal rows, 6–7.5–(8)/10 µm at the middle. Few short marginal striae rarely present between complete striae. A single continuous row of areolae runs along the valve mantle; mantle areolae aligned with the transverse striae. Rimoportulae one at each pole, positioned diagonally or on the same side of the apical axis. Rimoportulae with a round external pore, and an elongate slit-like internal opening. A reduced pseudocellus consisting of 3–7 fine pores at each valve end. Edge of the valve face ornamented by an irregular row of low siliceous dome-like papillae; presence of an extra row of finer papillae at the poles.

DERIVATION OF NAME: mi.ma.lli′s. femin., N.G. fem. *Mιμαλλίς* = a nymph, also another ancient name of Milos Island cited by Callimachus (310/305-240 BC).

HOLOTYPE: Botanical Museum of the Athens University, Greece; ADH slide 1317, England Finder Ref. L21/3; Figure 1A in this paper.

TYPE LOCALITY: Palaeochori Bay, Milos Island, Greece; marine, neritic, epipsammic.

SAMPLES EXAMINED: depth-zone-year = 4m-Υ-1997, 4m-W-1997, 7m-W-1996, 7m-W-1997, 7m-B-1997, 7m-C-1997.

***Meloneis mimallis* var. *zephyria* Louvrou & Econ.-Amilli var. nov.** Figure 1F

**Latin Diagnosis.** Valvae forma simillimae speciei *M. mimallis*, sed apicibus magis productis latitudine et rariora ordine linearum longarum areolarum, in medio 4–4.5/10 µm.

**Description.** Valves morphologicaly identical to the species *M. mimallis* but with broader produced apices and with loosely spaced longitudinal rows of areolae, 4–4.5/10 µm at the middle.

DERIVATION OF NAME: ze.phy.ri'.a. femin., N.G. femin. *Ζεϕυρία*, previous name of Milos island (according to Aristotle) possibly from the prevailing there western winds, Zephyros.

HOLOTYPE: Botanical Museum of the Athens University, Greece; ADH slide 1314, England Finder Ref. N47/0; Figure 1F in this paper.

TYPE LOCALITY: Palaeochori Bay, Milos Island, Greece

SAMPLES EXAMINED: depth-zone-year = 7m-W-1996.

***Meloneis akytos* Louvrou & Econ.-Amilli spec. nov.**  Figures 1G–H, 3C–E

**Latin Diagnosis.** Valvae planes facie, substrictis limbis, late lanceolatae, usque lateribus laeviter convexis et apicibus acriter productis; 21.5–32 µm longitudine, 14–19.5 µm latitudine. Area axialis hyalina, distincta, angusta, recta adusque mediocriter lanceolata. Areolae magnae circulares adusque ovatae velis rotoidibus dispositae in striis radialibus curvatis axialis areae. Areolae in striis radialibus, in medio 4.5–6 in 10 µm (in primis 5/10 µm), in margine 5–6.5/10 µm (in primis 6/10 µm), non frontales trans aream axialem. Areolae etiam dispositae in lineis minime curvatis in longitudinem, 5–6/10 µm in medio. Breves striae marginales praesentes inter perfectas strias. Una continua series areolarum trancurrit limbum valvae; areolae limbi continuae transversis striis. Rimoportulae, una in singulam apicem, locatae in diagonio vel in utram partem axis apicalis. Rimoportulae rotundo externali porello, et parte interna longa unica caesa. Valvae pseudocello extenuato ambobus apicibus. Pseudocelli se constant tribus et tribus an tribus et quattor (rariter duobus et duobus) porellis minutis per valvam. Margo valvae facies decorata irregulari serie demissarum silicearum papillarum sinilium cupolae forma.

**Description.** Valves with flat surface, shallow mantles, broadly lanceolate outline smoothly convex sides and acutely produced apices; length 21.5 to 32 µm, width 14 to 19.5 µm. Axial area hyaline, distinct, narrow, linear to slightly lanceolate. Transverse striae composed of very large circular to ovate areolae with rota-type vela, not aligned across the axial area. Transverse striae radial, in the middle 4.5–6/10 µm (mainly 5/10 µm), at the margin 5–6.5/10 µm (mainly 6/10 µm). Areolae arranged also in slightly curved longitudinal rows, 5–6/10 µm at the middle. Short marginal striae present between complete striae. A single continuous row of areolae runs along the mantle of the valve; mantle areolae aligned with the transverse striae. Rimoportulae one at each pole, positioned diagonally or on the same side of the apical axis. Rimoportulae with a round external pore, and an elongate slit-like internal opening. A reduced pseudocellus at each valve end consisting either of 3 and 3 or 3 and 4 (rarely 2 and 2) fine pores. Edge of the valve face ornamented by an irregular row of low siliceous dome-like papillae.

DERIVATION OF NAME: A'.ky.tos. femin., N.G. fem. *Άκυτος* = unlivable, unsuitable for residence; also, old name of Milos island.

HOLOTYPE: Botanical Museum of the Athens University, Greece; ADH slide 1318, England Finder Ref. T31/1; Figure 1G in this paper.

TYPE LOCALITY: Palaeochori Bay, Milos Island, Greece; marine, neritic, epipsammic.

SAMPLES EXAMINED: depth-zone-year = 4m-Υ-1997, 7m-W-1996, 7m-W-1997, 7m-B-1997.

***Meloneis gorgis* Louvrou & Econ.-Amilli sp. nov.**  Figures 1I–K

**Latin Diagnosis.** Valvae planes facie, substrictis limbis, late lanceolatae, usque lateribus laeviter convexis et apicibus acriter productis; 17–25 µm longitudine, 11–14 µm latitudine. Area axialis hyalina, distincta, recta et angusta ad polos valvae, et clariter latior in medio. Areolae circulares adusque ovatae velis rotoidibus dispositae in striis radialibus curvatis axialis areae. Areolae in striis radialibus, in medio 10–12/10 µm, in margine (8) 8.5–10/10 µm, non frontales trans aream axialem. Areolae etiam dispositae in lineis minime curvatis in longitudinem, (8.5) 9–10.5 (11)/10 µm in medio. Absentia brevium striarum marginalium inter perfectas strias. Una continua series areolarum trancurrit limbum valvae; areolae limbi continuae transversis striis. Rimoportulae, una in singulam apicem, locatae in diagonio vel in utram partem axis apicalis. Rimoportulae rotundo externali porello, et parte interna longa unica caesa. Valvae pseudocello extenuato ambobus apicibus. Pseudocelli se constant nonnullis porellis minutis per valvam. Margo valvae facies decorata irregulari serie demissarum silicearum papillarum sinilium cupolae forma.

**Description.** Valves with flat surface, shallow mantles, broadly lanceolate outline, smoothly convex sides and acutely produced apices; length 17 to 25 µm, width 11 to 14 µm. Axial area hyaline, distinct, linear and narrow at the valve ends and slightly widened at the middle. Transverse striae composed of circular to ovate areolae with rota-type vela, not aligned across the axial area. Transverse striae radial, in the middle 10–12/10 µm, at the margin (8) 8.5–10/10 µm. Areolae arranged also in slightly curved longitudinal rows, (8.5) 9–10.5 (11)/10 µm at the middle. Absence of short marginal striae between complete striae. A single continuous row of areolae runs along the mantle of the valve; mantle areolae aligned with the transverse striae. Rimoportulae one at each pole, positioned diagonally or on the same side of the apical axis. Rimoportulae with a round external pore, and an elongate slit-like internal opening. A reduced pseudocellus consisting of several fine pores at each valve end. Edge of the valve face ornamented by an irregular row of low siliceous dome-like papillae.

DERIVATION OF NAME: Gor.gi's. femin., N.G. fem. *Γοργίς* = old name of Milos island.

HOLOTYPE: Botanical Museum of the Athens University, Greece; ADH slide 1315, England Finder Ref. Q27/3; Figure 1I in this paper.

TYPE LOCALITY: Palaeochori Bay, Milos Island, Greece; marine, neritic, epipsammic.

SAMPLES EXAMINED: depth-zone-year = 4m-Y-1997, 7m-W-1996, 7m-W-1997, 7m-B-1997, 7m-C-1997.

## Discussion

Taxa of the order Rhaphoneidales are generally characterized by bipolar, multipolar or circular valves, areolae occluded by rotae, and rimoportulae usually present at the apices. The established genera of the family Rhaphoneidaceae, already mentioned in the introduction chapter, are distinguished by the type of valvar outline, type of areolae, number and position of rimoportulae, position and form of pseudocellus (apical pore field), alignment/nonalignment of the transverse rows of areolae across the axial area, presence/lack of spines or papillae, and presence/lack of surface furrows along the transapical striae [3], [5]–[7], [10]–[18], [20].

The detailed morphology of the new genus *Meloneis* is characterized by (i) valves broadly lanceolate with smoothly convex sides and slightly produced ends, forming an outline similar to that of *Adoneis*, some *Rhaphoneis*, and the fossil *Dickensoniaforma*, (ii) areolae circular or ovate, containing solid, simple rotae connected to the valve by two or scarcely three struts aligned parallel to the margin of the valve closest to the areola, a feature reminiscent of *Adoneis*, *Delphineis*, *Neodelphineis*, *Diplomenora*; areolae externally showing central pits like those of *Adoneis* and *Perissonoë*, (iii) transverse rows of areolae fully nonaligned across the axial area, as found in *Neodelphineis*, *Adoneis*, *Dickensoniaforma*, *Diplomenora*, *Detonia*, and some *Rhaphoneis*, (iv) a single row of areolae at the mantle below the marginal ridge in alignment with the transverse rows of areolae, a common feature of many genera such as *Delphineis*, *Neodelphineis*, *Perissonoë*, *Adoneis*, *Diplomenora*, and *Rhaphoneis* (at least *sensu* Round et al. 1990 [1]), (v) rimoportulae one at each pole, diagonally or laterally positioned in relation to the longitudinal axis and lying at the last transverse row of areolae, as observed in *Neodelphineis*, *Perissonoë* (with 1–4 rimoportulae per valve), *Rhaphoneis* (?), and *Lancineis* (?), (vi) pseudocelli one at each pole, consisting of two to seven very fine pores in a rather circular pattern between the last transverse row of areolae and the marginal ring of papillae, a feature unique for *Meloneis* and vaguely similar to those of *Neodelphineis* (with one pore), *Perissonoë* (with pores in a rather radiating pattern), and *Rhaphoneis* and *Lancineis* (with many pores in a rather disorganised pattern), (vii) lack of surface furrows along the transapical striae, similarly to the genera *Adoneis*, *Detonia*, *Dickensoniaforma*, *Diplomenora*, *Lancineis*, *Perissonoë*, *Sceptroneis* and most possibly *Rhaphoneis*, in contrast to the genera *Drewsandria*, *Neodelphineis* and most *Delphineis* having grooved external valve face (the valves of the *Delphineis surirella* group are usually smooth or very slightly grooved [6], [31]); (viii) presence of a ring of papillae at the valve margin, a feature also found in *Perissonoë*, some *Delphineis*, and partly in *Adoneis* (*Neodelphineis* bears short spines).

The above descriptive comparison shows a closer similarity of *Meloneis* to the genera *Rhaphoneis*, *Neodelphineis*, and *Perissonoë* especially concerning the fine structure of the apex (Table 1).

Concerning genus *Rhaphoneis sensu lato* (see [32]) there is a lack of knowledge about the fine structure of some taxonomic features in several species. Therefore, comparison can be made to *Rhaphoneis amphiceros* (Ehrenberg) Ehrenberg [18], [19], the type species of the genus in which certain rhombic or broadly lanceolate forms were attributed as synonyms (i.e. *R. rhombus* (Ehrenberg) Ehrenberg, *R. amphiceros* var. *rhombica* Grunow in Van Heurck, *R. amphiceros* f. *minor* Grunow in Van Heurck) or remained as valid taxa [i.e. *R. amphiceros* var. *amazonica* (Grunow in Pantocsek) M.Peragallo, *R. amphiceros* var. *gemmifera* (Ehrenberg) Peragallo & Peragallo]. Besides, some drawings of *R. amphiceros* var. *rhombica* depicted later by several authors (e.g. [33] p. 29, fig. 10/17–19; [34] p. 329, fig. 83/22–23; and [35] p. 100, fig. 5/7 as *R. rhombica* Andrews), as well as the variety *R. amphiceros* var. *intermedia* (Pantocsek) M.Peragallo and the species *R. debyi* Pantocsek and *R. subtilissima* Pantocsek, are reminiscent in general appearance of *Meloneis*. Generally, in *R. amphiceros* the areolae are occluded by perforated rotae, the pseudocellus is composed by many pores in a rather non-oriented pattern and there is absence of papillae or spines [1]; additionally, it seems that the single row of marginal areolae on the mantle of the valve is not continuous around the apices. However, within *R. amphiceros* there were also classified some discrepant individuals clearly having some valve structure characteristics of *Meloneis* (e.g. [36] p. 51, fig. 9/2; [37] p. 101, fig. 8/5; [38] figs 16 & 47).
Although *Neodelphineis* and *Perissonoë* have a different outline (elongate to elliptical valves, and quadrate or triangular respectively) from that of *Meloneis*, the three genera show similarities in the position of both pseudocelli and rimoportulae. However, in *Perissonoë* there are many pores in an almost radiating pattern, in *Neodelphineis* there is a reduction of the number of fine pores to one, whereas *Meloneis* with a rather circular pattern of 2 to several pores keeps an intermediate position between them concerning this particular feature.
Similarities of *Meloneis* to other genera such as *Adoneis* and *Dickensoniaforma* are considered as rather superficial since they are restricted mainly to the general valve appearance and outline. Specifically, genus *Adoneis* bears an apical rimoportula at each pole positioned among the pores of pseudocellus, and additional rimoportulae at each lateral valve margin; genus *Dickensoniaforma* lacks pseudocelli and the apical pore fields, ocelli, appear to be consiting of vestigial (?) areolae i.e. fine areolae.

**Table 1:** Valve morphology - according to the available literature and photodocumentation cited in this paper - differentiating *Meloneis* from the related genera of the family Rhaphoneidaceae (for the genus *Rhaphoneis s.s.* characters of the type species *Rhaphoneis amphiceros* were considered).

Related Genera of Rhaphoneidaceae	*Meloneis*	*Neodelphineis*	*Perissonoë*	*Rhaphoneis s.s.*
Specimen Status^1^	Re	Re	Re, Fo	Re, Fo
Valve Outline^2^	La	Li, Elo-Ell	Mu	Rh
Rota of the areolae^3^	So	So	Pe	Co
Mantle areolae^4^	SRa	SRa	SRa	SRna
Number of Rimoportulae	2	2	1–4	2
Types of Apical Pore Field^5^	Ps	RP_1_	Ps	Ps
Number of pores in Apical Pore Field^6^	F	1	M	M
Pattern of pores in Apical Pore Field^7^	Ci		Rd	Di
Position of Apical Rimoportula and Apical Pore Field^8^	vE→ Ps→ (R-Lr)	vE→ RP_1_→ (R-Lr)	vE→ Ps→ (R-Lr)	vE→ Ps→ ? R→ ? Lr
External furrows along the transapical striae^9^	A	P	P, A	A
Protrusions^10^	Pa	Sp	Pa	A

1 Fo = fossil, R = recent.

2 Mu = multipolar, La = lanceolate, Rh = rhomboid, Ell = elliptic, Li = linear, Elo = elongate.

3 So = solid, Pe = perforate, Co = concentric.

4 SRa = presence of a single row of areolae even around the apices, SRna = presence of a single row of areolae but not around the apices.

5 Ps = pseudocellus, RP_1_ = apical pore field reduced to 1 pore.

6 x = number, M = many, F = few.

7 Ci = rather circular, Di = rather disorganised, Rd = rather radiating.

8 Rimoportula (R) and Apical Pore Field (Ps, RP_1_) in relation to vE = valve edge, and in relation to Lr = last transverse row of areolae. For instance: (R-Lr) = rimoportula positioned between the areolae of the last transverse row, vE→ Ps = pseudocellus positioned next to the valve edge. The question marks indicate unclear position of the rimoportula in relation to pseudocellus and to the last transverse row.

9 P = presence, A = absence.

10 Sp = spines, Pa = papillae, A = absence of protrusions.

Therefore, the unique combination of features cited above and especially the morphology of pseudocellus and papillae suggest a new taxon deserving a distinct taxonomic status, i.e. *Meloneis* gen. nov. Within genus *Meloneis*, three species and one variety (i.e. *M. mimallis*, *M. mimallis* var. *zephyria*, *M. akytos*, *M. gorgis*) are discernible (see Table 2) mainly differing in size and polar outline of the valves, size of areolae, pore number in pseudocelli, and densities of transapical striae and areolae.

**Table 2:** Characters of valve morphology differentiating the four taxa of *Meloneis*.

Taxon	valve length (µm)	valve width (µm)	valve apices	transverse striae	striae density (in 10 µm)	areolae	areolae density (in 10 µm)	pores of pseudocellus
*Meloneis mimallis* var. *mimallis*	21.5–29	14.5–18.5	rounded, slightly produced	radial	(7.5) 8–8.5 (9)	large	6–7.5 (8)	3–7
*Meloneis mimallis* var. *zephyria*	26.5–27	17.5–18	rounded, broadly produced	noticeably radial	7.5	large, loosely spaced	4–4.5	not seen
*Meloneis akytos*	21.5–32	14–19.5	acutely produced	noticeably radial	4.5–6	very large, loosely spaced	5–6	2–4
*Meloneis gorgis*	17–25	11–14	acutely produced	radial	10–12	small	(8.5) 9–10.5 (11)	not seen
